# Effects of task context on EEG correlates of mind-wandering

**DOI:** 10.3758/s13415-023-01138-9

**Published:** 2023-11-29

**Authors:** Rebecca J. Compton, Danylo Shudrenko, Katelyn Mann, Emil Turdukulov, Erin Ng, Lucas Miller

**Affiliations:** Psychology Department and Neuroscience Program, Haverford College, Haverford, PA USA

**Keywords:** Mind-wandering, Attention, EEG, Alpha oscillations

## Abstract

This study was designed to examine how mind-wandering and its neural correlates vary across tasks with different attentional demands, motivated by the context regulation hypothesis of mind-wandering. Participants (*n* = 59 undergraduates) completed the sustained attention to response task (SART) and the Stroop selective attention task in counterbalanced order while EEG was recorded. The tasks included experience-sampling probes to identify self-reported episodes of mind-wandering, along with retrospective reports. Participants reported more mind-wandering during the SART than the Stroop and during whichever task was presented second during the session, compared with first. Replicating previous findings, EEG data (*n* = 37 usable participants) indicated increased alpha oscillations during episodes of mind-wandering, compared with on-task episodes, for both the SART and Stroop tasks. ERP data, focused on the P2 component reflecting perceptual processing, found that mind-wandering was associated with increased P2 amplitudes during the Stroop task, counter to predictions from the perceptual decoupling theory. Overall, the study found that self-report and neural correlates of mind-wandering are sensitive to task context. This line of research can further the understanding of how mechanisms of mind-wandering are adapted to varied tasks and situations.

**Supplementary Information:**

The online version contains supplementary material available at 10.3758/s13415-023-01138-9.

## Introduction

As the study of mind-wandering has increased over recent decades, an unresolved issue is the extent to which mind-wandering, defined as “off-task thinking” or attention to internal thoughts rather than external stimuli or tasks, should be considered an adaptive or maladaptive process (McVay & Kane, 2010; Mooneyham & Schooler, 2013; Smallwood & Schooler, 2015). Mind-wandering may reflect a lapse or failure of attentional control, when our thoughts drift even as we “should be” attending to some external task, such as listening to a class lecture or driving a car. Alternatively, mind-wandering may reflect an adaptive allocation of attention, depending on the circumstances. Internal thoughts may be more relevant to an individual’s personal goals compared with a task imposed externally. For example, engaging in internal problem-solving about a social conflict may be more important to a person than pressing buttons in a laboratory task, in which case mind-wandering during the task could be adaptive in the broader context of personally relevant goals. Additionally, with effective attentional allocation, people may manage to mind-wander while maintaining acceptable task performance, effectively meeting two sets of goals.

The context regulation theory of mind-wandering (Smallwood & Andrews-Hanna, 2013; Smallwood & Schooler, 2015) proposes that people regulate their mind-wandering to suit the current context. For example, when an external task is easy, people are more likely to mind-wander than when the task is difficult. Presumably, the easy external task does not require as much attentional capacity; therefore, more attentional resources are available to expend on internally directed thinking without negative consequence for the external task performance. Indeed, mind-wandering tends to be more frequent during less-demanding tasks (Rummel & Boywitt, 2014; Robison et al., 2020). A meta-analysis found that the effect of mind-wandering on performance was reduced for easy compared with more complex external tasks (Randall et al., 2014).

Another approach to the question of adaptive versus maladaptive mind-wandering is to consider that mind-wandering comes in different varieties (Seli et al., 2016, 2018). Deliberate mind-wandering, in which a person intentionally allows their thoughts to drift, may be more adaptive than spontaneous mind-wandering, in which the thoughts drift even without intention. Spontaneous mind-wandering, but not deliberate mind-wandering, is associated with negative affect (Seli et al., 2019) and with conditions such as attention-deficit disorder and obsessive-compulsive disorder (Seli et al., 2015). Thus, perhaps deliberate, intentional mind-wandering is more reflective of adaptive attentional control, whereas spontaneous mind-wandering reflects a maladaptive control failure.

This study was designed to address questions related to the modulation of mind-wandering by using neural measures that are sensitive to internal states of mind-wandering during an externally imposed cognitive task (Christoff et al., 2016; Gruberger et al., 2011; Smallwood et al., 2021). Previous studies have found that episodes of mind-wandering are correlated with activity in the brain’s default mode network in fMRI studies (Andrews-Hanna et al., 2010; Christoff et al., 2009), with reduced neural responses to external stimuli as indexed by event-related potential (ERP) responses (Barron et al., 2011; Kam & Handy, 2013; Smallwood et al., 2008), and with increased EEG oscillations in the alpha frequency range (8–12 Hz), thought to reflect internally directed thinking (Arnau et al., 2020; Compton et al., 2019; da Silva et al., 2022). However, little research has examined the contextual modulation of these neural markers of mind-wandering or their association with different varieties of mind-wandering experience, such as deliberate versus spontaneous mind-wandering. The present study attempts to fill this gap.

First, we propose that neural correlates of mind-wandering should track predictions from the context regulation hypothesis. In general, task contexts that produce more mind-wandering should produce increased neural indices of mind-wandering. The present study uses EEG/ERP methods to address these questions. As noted above, previous studies have found that mind-wandering is associated with increased alpha oscillations in the EEG (Kam et al., 2022), consistent with the idea that alpha waves index internally directed thought (Bowman et al., 2017) and the suppression of external visual processing (Clayton et al., 2018). We predict that a less attentionally demanding task should be associated with both increased self-reported mind-wandering and increased alpha oscillations compared with a more attentionally demanding task. Likewise, other studies have reported reduced ERP responses to external stimuli while mind-wandering (Kam et al., 2022), consistent with a theory that perception is “decoupled” from the external environment when the mind wanders (Smallwood et al., 2008). We propose that this ERP perceptual-decoupling effect also should be context-dependent. In less-demanding task contexts in which mind-wandering is more likely, the perceptual decoupling that accompanies mind-wandering should be enhanced.

To test these predictions, we used two tasks that differ in their attentional demands. The first task—the sustained-attention-to-response task (SART; Robertson et al., 1997)—has been used in many studies of mind-wandering, because it involves low attentional demands and thus commonly induces mind-wandering (McVay & Kane, 2009; Leszczynski et al., 2017; Mrazek et al., 2012a, b; Seli et al., 2016; Wiemers & Redick, 2019). In the SART, participants view a series of stimuli, most of which require a simple keypress response. Embedded in the series are periodic “no-go” stimuli, to which the participant must withhold their response. Accuracy on the no-go trials—successfully inhibiting a response—serves as the primary index of performance.

In addition to the SART, we also implemented a challenging version of the classic Stroop task of selective attention (Stroop, 1935), in which participants indicate via keypress the font color of a word while ignoring its meaning. Our version of the Stroop task was made difficult by the use of six different colors, mapped onto to six different keys on the keyboard. Thus, participants must selectively ignore word meaning, which is sometimes incongruent with font color (e.g., RED in blue font). They also must remember stimulus-response mappings for the six font colors. Although mind-wandering occurs during the Stroop task and is accompanied by increased alpha activity (Compton et al., 2019), no studies to date have directly compared mind-wandering and its neural correlates between the more-demanding Stroop task and the less-demanding, but more frequently studied, SART.

Our primary predictions include both within-task and across-task effects. Within each task, we expect that when participants report mind-wandering versus being on-task in response to periodic experience-sampling probes, they will exhibit increased alpha oscillations and decreased ERP responses to external stimuli, consistent with the perceptual decoupling theory. These predictions are conceptual replications of findings from previous studies, applied to two different tasks to increase generalizability. In addition, the context regulation theory predicts that the SART should produce more self-reported mind-wandering than the Stroop task, because the SART is less attentionally demanding. Our study addresses whether the SART also will be characterized by increased alpha oscillations and greater ERP-indexed perceptual decoupling than the Stroop task.

Because our study asked participants to complete two fairly long and repetitive attention tasks, we must consider whether the timing of a task during the session affects mind-wandering and its neural correlates. Mind-wandering is associated with boredom (Raffaelli et al., 2018) and low alertness (Robison et al., 2020), both of which seem more likely for whichever task is presented second in the sequence. Furthermore, previous studies have found that mind-wandering increases with task duration (Risko et al., 2012; Thomson et al., 2014). Thus, we examined whether mind-wandering and its neural correlates are more evident in the second task of the session, with the order of the two tasks counterbalanced across participants.

Finally, in addition to testing the context regulation hypothesis using these two tasks, we sought to address potential correlates of deliberate versus spontaneous mind-wandering. Starting from the premise that deliberate mind-wandering indicates adaptive cognitive control, we predicted that individuals who report greater deliberate (versus spontaneous) mind-wandering should exhibit better task performance. We also predicted that deliberate mind-wandering should be correlated with an independent self-reported measure of executive functioning. On a more exploratory basis, we hypothesized that participants who tend to mind-wander deliberately also would tend to exhibit more dynamic modulation of neural measures across task contexts. Although exploratory, this final hypothesis is consistent with previous evidence that individuals who tend to exhibit more spontaneous rather than deliberate mind-wandering did not modulate mind-wandering or EEG alpha power as consistently across tasks (Bozhilova et al., 2021, 2022).

In sum, the present study addressed a set of predictions pertaining to the modulation of mind-wandering and its neural correlates across task contexts. In a study design involving two tasks with different attentional demands, we predicted that mind-wandering and its EEG and ERP correlates would be enhanced for the easier of the two tasks. Furthermore, we explored whether individual differences in the degree of self-reported deliberate mind-wandering would predict contextual modulation of mind-wandering and its EEG/ERP correlates.

## Method

### Participants

Fifty-nine undergraduates were recruited from the Haverford College community through flyers and electronic postings. All procedures were approved by the Haverford College Institutional Review Board.

Participants ranged in age from 18 to 22 years and included 21 self-identified men, 33 women, four nonbinary individuals, and one who declined to state a gender. With regard to ethnicity, 40 described themselves as white/Caucasian, six as Hispanic/Latino, 18 as Asian/Pacific Islander, two as black/African-American, one as Native American, and two chose to specify an alternative label (North African, South Asian). Numbers add up to more than 100% of the sample because ten of the participants selected more than one race/ethnicity.

Among the 59 participants, some datasets could not be fully utilized for various reasons. All participants had adequate self-report data. Participants were excluded from performance analyses if their accuracy was more than 3 standard deviations below the mean for the task, indicating a failure to understand or implement task instructions. This criterion resulted in the exclusion of two participants from the SART and one participant from the Stroop task. Finally, EEG data from eight participants could not be used because a hardware problem resulted in event markers not registering with the EEG data. Additionally, EEG data from three participants could not be used because a faulty VEOG connection precluded the appropriate removal of blink artifacts. Therefore, only 48 participants had usable EEG data. As described further below, analyses of EEG data pertaining to mind-wandering versus on-task episodes were further limited, because some participants reported a very low number of either of these episode types. Thus, key analyses of EEG data focused on a subset of 37 participants who had adequate instances of both types of episode (mind-wandering and on-task) in both the SART and the Stroop. This sample size compares favorably with previous studies of mind-wandering using EEG methods (see systematic review in Kam et al., 2022, Tables 1 and 2).

**Table 1:** Descriptive statistics for self-report measures of mind-wandering (MW)

	Mean (SD)	Range (min-max)
SART probe count	7.03 (3.01)	0–14
Stroop probe count	6.44 (3.66)	0–15
SART MW % estimate	54.8 (25.3)	0–100
Stroop MW % estimate	44.6 (28.3)	0–100
SART deliberate % estimate	33.4 (26.1)	0–100
Stroop deliberate % estimate	27.5 (25.1)	0–90
Retrospective – MW %overall	52.6 (22.7)	10–92
Retrospective – MW %deliberate	32.3 (25.0)	0–90
Retrospective – MW %spontaneous	59.5 (28.0)	10–100

*N* = 59

**Table 2:** Correlation matrix including all self-report measures of mind-wandering (MW)

	1. SART probe count	2. Stroop probe count	3. SART MW%	4. Stroop MW%	5. SART delib. MW%	6. Stroop delib. MW%	7. Retro MW overall	8. Retro MW delib.	9. Retro MW spont.
1.	--								
2.	0.44***	--							
3.	0.77***	0.42***	--						
4.	0.36**	0.74***	0.48***	--					
5.	0.29*	-0.01	0.20	0.01	--				
6.	0.13	0.08	0.16	0.14	0.41**	--			
7.	0.64***	0.63***	0.71***	0.57***	0.05	0.01	--		
8.	0.27*	0.03	0.22	-0.05	0.64***	0.57***	0.25	--	
9.	-0.08	-0.01	-0.04	0.08	-0.46***	-0.55***	0.03	-0.65***	--

Entries in the table are Pearson’s correlation coefficients. * *p* < .05, ** *p* < .01, ***p < .001; *N* = 59

### Procedure overview

Following informed consent, the participant was fitted with the EEG cap and then completed the two cognitive tasks, controlled by E-prime software, while EEG was recorded. The E-prime program randomized whether the participant completed the SART or Stroop task first (*n* = 31 SART first, *n* = 28 Stroop first). The second task followed immediately after the first. After the two cognitive tasks, EEG recording was stopped and the participant then completed the post-task questionnaire at the same computer as the cognitive tasks. After the questionnaire, the EEG cap was removed and the participant was paid and debriefed.

### SART

The instructions for the SART indicated that participants would see single digits displayed on the screen, one at a time, and should press the spacebar for every digit except 3. “Go” trials included the digits 0–2 and 4–9, whereas “no-go” trials refer to the digit 3. Digits were presented in white against a black background. Trials were organized into 3 blocks of 150 trials each (15 repetitions of each digit within each block, with order randomized), resulting in a total of 405 go trials and 45 no-go trials across the 3 blocks. Between blocks, participants had the opportunity to rest their eyes briefly before continuing with a press of the spacebar key. Digits were presented for a maximum of 1000 ms or until termination by a keypress, and trials were separated by a 1000-ms intertrial interval during which the screen was black. There were no practice trials for the SART because the stimulus-response mapping was very simple.

### Stroop task

In the Stroop task, participants had to press one of six keys to indicate the font color in which a stimulus word was printed. The colors red, orange, yellow, green, blue, and purple were mapped onto six keys corresponding to the first three fingers of each hand in standard keyboard typing position. The task began with a practice set of 12 trials, in which participants received trial-by-trial accuracy feedback, in order to learn the stimulus-response mapping. After the practice set, the participant completed 3 sets of 120 trials each. Half the trials within each set were color-word congruent (e.g., BLUE in blue font) and half were color-word incongruent (e.g., BLUE in red font). All words were presented against a black background. Each of 12 unique stimuli (6 congruent and 6 incongruent) was represented ten times within the set of 120, in randomized order. Across all three blocks, the total number of trials included 180 congruent and 180 incongruent trials. Word stimuli were presented for 250 ms, followed by a blank screen that terminated upon keypress, followed by a 1280-ms interval before the next stimulus. Participants were given the opportunity to rest their eyes briefly between blocks before continuing.

### Mind-wandering probes

During both the SART and Stroop tasks, five experience-sampling probes occurred randomly within each block (15 total probes per task). The probe screen asked the participant to indicate with a keypress whether their mind was on-task or wandering just prior to the probe. These concepts were defined at the beginning of the task as follows: “On-task means that your thoughts are fully focused on the task itself. Mind-wandering means that your thoughts have wandered away from the task and you are thinking about something else, like plans later in the day or something that happened yesterday. As long as you are thinking about something else, choose the mind-wandering option rather than saying you are on-task.”

In addition, at the end of each task, the program asked the participant to indicate, on a sliding scale (0–100%), what percent of time they believed their mind was wandering during the task they just finished. Then, the program presented a second sliding scale that asked what percent of their mind-wandering was deliberate, defined as “you let your mind wander intentionally to other thoughts because you were bored or wanted to think about something else. The opposite of deliberate is spontaneous or involuntary mind-wandering, when your mind drifts even though you don’t intend for it to do so, or even when you are trying to pay attention to the task.” On the sliding scale, 0% was labeled as “NONE of your mind-wandering was deliberate; ALL of it was spontaneous or unintentional” and 100% was labeled as “ALL of your mind-wandering was deliberate; NONE of it was spontaneous or unintentional.”

### Post-task questionnaire

After both cognitive tasks, the participant completed a questionnaire implemented in Qualtrics. The questionnaire included the following sections, presented in the same order for all participants, followed by demographic items including age, gender, and ethnicity.

Single-item questions about mind-wandering across both tasks: (1) What percentage of the time during the tasks do you think your mind was wandering, as opposed to being focused on the task? (0–100); (2) What percentage of your mind-wandering do you think was deliberate, meaning that you engaged in mind-wandering intentionally in order to think about something else instead of the task (e.g., make plans for later, solve a problem, think back on a prior experience with intention, avoid boredom of the task)? (0–100); (3) What percentage of your mind-wandering do you think was unintentional, meaning that your mind kept being distracted by other thoughts even when you were trying to focus on the task? (0–100)

#### Mind-Wandering Questionnaire (MWQ; Mrazek et al., 2013)

Participants rated their agreement with five statements, such as “I have difficulty maintaining focus on simple or repetitive work” on a 6-point scale (*almost never* to *almost always*).

#### Behavioral Rating Inventory for Executive Functions-Adult Version (BRIEF; Roth & Gioia, 2005)

This standardized instrument is a 75-item, self-report survey that asks participants to indicate the extent to which they experience challenges in certain aspects of thinking and behavior (e.g., “I don’t plan ahead for future activities”; “I make careless mistakes”; “I have difficulty finishing a task on my own”). Responses are given on a 3-point scale (1 = *never*, 2 = *sometimes*, 3 = *often*). Scoring involves tallying responses for each of nine subscales (inhibit, shift, emotional control, self-monitor, initiate, working memory, plan, task monitor, and organization) as well as a global composite that sums across the nine subscales. Only the global composite was examined for purposes of this study. Higher scores indicate greater self-endorsement of problems in executive functions.

### EEG data acquisition

EEG data were acquired continuously using a Compumedics neo-net cap system and Grael amplifier controlled by Curry software. We recorded from scalp sites arranged in a 3 x 3 grid, including F3, Fz, F4, C3, Cz, C4, P3, Pz, P4, in addition to left and right mastoid sites. All sites were referenced to a central location at the time of acquisition. Vertical and horizontal eye channels were calculated by using electrodes positioned above and below the left eye and on the left and right temples and were computed as two bipolar pairs by the Grael amplifier. The sampling rate was 2048 Hz. The Grael amplifier received event markers from E-prime via the Chronos timing device.

### EEG data processing

Offline preprocessing of raw data files involved applying a 1–30 Hz filter, re-referencing sites to the average of the two mastoids (with the exception of one participant with a bad left mastoid, for whom data were re-referenced only to the right mastoid), and applying Curry’s spatial filter algorithm to remove blink artifacts. For three participants with HEOG artifact, the spatial filter was also applied to removed that artifact from scalp channels. Visual inspection of individual data files was conducted to exclude segments with any remaining gross artifact and subsequently any epochs with voltages exceeding ±150 microvolts were excluded.

To address hypotheses related to oscillatory activity, epochs were created 5 s before experience sampling probes, sorted according to whether the participant indicated mind-wandering (MW) or being on-task (OT) in response to the probe. The 5-s interval was selected based on previous research that found MW versus OT alpha effects with that interval length (Compton et al., 2019). A power spectrum was created for each epoch by using the fast Fourier transform implemented in Curry (applying a Hann 10% taper). Spectra were then averaged across 5-s epochs separately for each probe-response type (MW and OT). Finally, power in the alpha frequency range (8–12 Hz) was extracted from the average power spectrum for each probe-response type. Participants were included in this analysis only if they had at least three instances of mind-wandering and at least three instances of being on-task during both the SART and the Stroop (*n* = 37; mean number of epochs per response type = 7.5).

To calculate ERPs, the continuous file was epoched around event markers for both the SART and the Stroop, from −200 to 800 ms surrounding the stimulus onset. Epochs were averaged separately by trial type (go and no-go for the SART; congruent and incongruent for the Stroop) to confirm that typical task effects in the ERPs were observed.

Subsequently, epochs were sorted and averaged according to their relationship to the probe response. OT epochs included those five trials before a probe to which the participant reported being on-task. MW epochs included those five trials before a probe to which the participant reported mind-wandering. For the OT and MW trials in the SART, only go trials were included due to the infrequency of no-go trials. For the OT and MW trials in the Stroop, both congruent and incongruent trials were included. As for the alpha analysis, participants were included in this analysis only if they had at least three instances of mind-wandering and at least three instances of being on-task during both the SART and the Stroop (*n* = 37). The average number of trials contributing to ERP waveforms was 34.2 trials for SART-MW, 40.8 trials for SART-OT, 33.1 trials for Stroop-MW, and 41.9 trials for Stroop-OT.

From these ERP waveforms, P2 peak amplitudes were selected on the basis of visual inspection of grand-average waveforms (see Fig. 4 in *Results* section). P2 peak amplitudes for each participant, trial type, and site were quantified by Curry software, with the peak defined as the most positive point between 100 and 250 ms after stimulus onset. Although previous studies have examined the P3 amplitude in relation to mind-wandering (Kam et al., 2022), we did not quantify the P3 peak in the MW-OT analysis, because visual inspection of the waveforms did not suggest a clearly identifiable P3 peak in the go-trial and Stroop waveforms.

## Results

### Self-report measures of mind-wandering

Descriptive statistics for all self-report measures of mind-wandering are presented in Table 1. On average, participants estimated mind-wandering 54.8% of the time during the SART, 44.6% during the Stroop, and 52.6% in the overall retrospective estimate. Likewise, responses to the in-task probes indicated that participants reported mind-wandering approximately half the time (6–7 mind-wandering responses to the 15 probes).

The multiple measures of mind-wandering were generally intercorrelated with one another (Table 2). However, estimates of deliberate mind-wandering tended to correlate only with other measures of deliberate mind-wandering (and negatively with spontaneous mind-wandering), rather than with overall mind-wandering estimates. This pattern was confirmed by a principal components analysis (varimax rotation) of self-report mind-wandering measures, which revealed two components (Table 3). The first component (accounting for 36.9% of variance) had high loadings from all general mind-wandering measures, and the second (accounting for 30.2% of variance) had high loadings from questions pertaining to deliberate versus spontaneous mind-wandering. These analyses suggest two orthogonal dimensions of individual differences in mind-wandering, one indexing overall tendency to mind-wander and another indexing the tendency to mind-wander deliberately versus spontaneously. Component scores for individual participants were extracted and used to simplify correlations among performance, self-report, and EEG variables in subsequent analyses.

**Table 3:** Principal components analysis of self-reported mind-wandering variables

	Component 1 loading (general mind-wandering)	Component 2 loading (deliberate mind-wandering)
SART probe count	0.772	—
Stroop probe count	0.796	—
SART MW % estimate	0.821	—
Stroop MW % estimate	0.782	—
SART deliberate % estimate	—	0.774
Stroop deliberate % estimate	—	0.748
Retrospective – MW %overall	0.882	—
Retrospective – MW %deliberate	—	0.886
Retrospective – MW %spontaneous	—	−0.817

Loadings below 0.3 are represented by —

The next set of analyses addressed whether the amount of mind-wandering differed between the SART and Stroop tasks and whether the amount of mind-wandering depended on task order. We conducted 2 x 2 mixed-factorial ANOVAs with the within-subjects factor Task (SART, Stroop) and the between-subjects factor Task Order (SART first/Stroop second, Stroop first/SART second). This analysis was conducted for three separate dependent variables: mind-wandering probe count, % time estimated mind-wandering, and % time estimated deliberately mind-wandering.

**Fig. 1:**
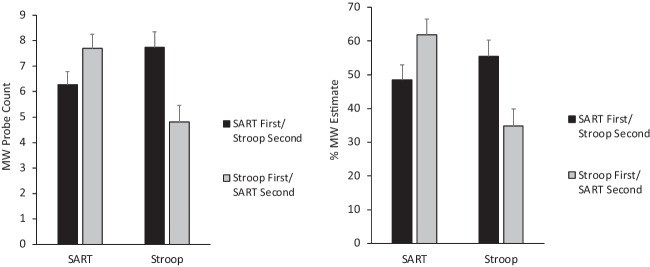
Self-reported mind-wandering (MW) during the SART and Stroop tasks, separated according to which task was completed first. *Note*: Error bars are ± standard error of the mean

For the probe count variable, the ANOVA revealed a marginal main effect of Task (*F*(1, 57) = 3.47, *p* < .07), no main effect of Task Order (*p* > .50), and a significant Task x Task Order interaction effect (*F*(1, 57) = 31.71, *p* < .001). Means for the interaction are displayed in Fig. 1 (left panel). The crossover pattern in the interaction reflects more mind-wandering responses to probes in whichever task was presented second, compared to when that task was presented first. That is, there was more mind-wandering during the SART when the SART was presented second rather than first (*t*(57) = 2.15, *p* = .036); likewise, there was more mind-wandering during the Stroop when the Stroop was presented second rather than first (*t*(57) = −2.90, *p* = .005). The marginal main effect of Task reflected the fact that collapsing across order effects, mind-wandering responses to the probes tended to be slightly higher overall during the SART (*M* = 7.08) than the Stroop (*M* = 6.37).

For the estimate of % time spent mind-wandering, the main effect of Task (*F*(1, 57) = 17.1, *p* < .001) reflected overall more mind-wandering during the SART (*M* = 55.2%) than the Stroop (*M* = 44.0%). However, this was qualified by the significant Task x Task Order interaction (*F*(1, 57) = 44.9, *p* < .001), whose means are displayed in Fig. 1 (right panel). Similar to the pattern for the probe count variable, mind-wandering during a given task was greater when that task was presented second rather than first. That is, mind-wandering during the SART was higher when the SART was second rather than first (*t*(57) = 2.41, *p* = 0.019), and mind-wandering during the Stroop was higher when the Stroop was second rather than first (*t*(57) = −3.03, *p* = .004). The main effect of Task Order was not significant (*p* > .60).

Results for the estimate of % time deliberately mind-wandering revealed a different pattern, in which the only significant effect was a main effect of Task Order (*F*(1, 57) = 6.20, *p* = .016). Means revealed greater estimated deliberate mind-wandering for the Stroop-first/SART-second participants (*M* = 37.6%) than for the SART-first/Stroop-second participants (*M* = 24.0%). However, there was no significant main effect of Task for the % of deliberate mind-wandering (*p* > .10), and the interaction was not significant (*p* > .30).

In sum, mind-wandering depended on task context factors for all three dependent variables. Overall mind-wandering tended to be reported more for the SART than the Stroop task and more for the second task compared with the first task. Both of these patterns are consistent with the context regulation hypothesis. Self-reports of deliberate mind-wandering also were affected by task order.

### Task performance

On the SART, mean accuracy was 99.1% correct on go trials and 62.4% correct on no-go trials, which required a response inhibition. Correlations between mind-wandering and SART performance found that lower accuracy on no-go trials was correlated with the component reflecting overall mind-wandering (*r* = −0.360, *p* = .006) but not with the component reflecting deliberate mind-wandering (*p* > .40). Neither component was associated with accuracy on go trials, which is not surprising given that go-trial performance was near ceiling levels.

On the Stroop task, mean accuracy was 90.8% correct for congruent and 87.0% correct for incongruent trials (paired t-test, *t*(57) = 6.80, *p* < .001), consistent with an overall Stroop interference effect. Likewise, responses were slower on incongruent trials (*M* = 972 ms) than congruent trials (*M* = 836 ms; paired *t*-test, *t*(57) = −5.46, *p* < .001). Scores for the general mind-wandering component were correlated with significantly lower accuracy (*r* = −0.312, *p* = .017) and marginally slower reaction time (*r* = 0.251, *p* = 0.058), whereas deliberate mind-wandering component scores did not predict task performance (*p*s > .30).

To confirm the assumption that the SART and Stroop differ in overall difficulty level, we compared mean reaction times between the SART go trials and Stroop trials (including both congruent and incongruent trial types). As expected, overall responses were significantly slower on the Stroop (*M* = 908 ms) compared with the SART (*M* = 341 ms; *t*(56) = −10.3, *p* < .001). Likewise, overall task accuracy was lower for the Stroop (*M* = 89.8% correct) than the SART (*M* = 95.4% correct across Go/No-Go trials; *t*(56) = -3.30, *p* < .002).

In sum, the performance data indicated overall patterns expected for the tasks, including high commission errors on the SART and typical Stroop congruency effects. Comparisons between tasks confirmed that performance was slower and less accurate on the Stroop compared with the SART, supporting the assumption that the Stroop task is more difficult. In addition, participants who reported more mind-wandering tended to perform worse on both tasks, whereas individual differences in the extent of deliberate mind-wandering were not associated with task performance.

### Self-reported executive function

We next examined whether the key measure of self-reported executive function—the BRIEF global composite score—was correlated with mind-wandering self-reports and task performance. Higher BRIEF scores (indicating more executive functioning challenges) were positively associated with the component indexing overall mind-wandering, but the correlation did not reach significance (*r* = 0.216, *p* = 0.10). Contrary to prediction, BRIEF scores were not associated with the deliberate mind-wandering component scores (*r* = −0.152, *p* = 0.25). The BRIEF scores were associated with slower reaction times on the Stroop task (*r* = 0.316, *p* = .016) but not accuracy on the Stroop task (*r* = −0.011, *p* = 0.939) and were not significantly correlated with accuracy on SART no-go trials (*r* = −0.209, *p* = .118). In sum, self-reported executive functioning challenges were not strongly associated with mind-wandering or task performance.

### Oscillatory EEG data

**Fig. 2:**
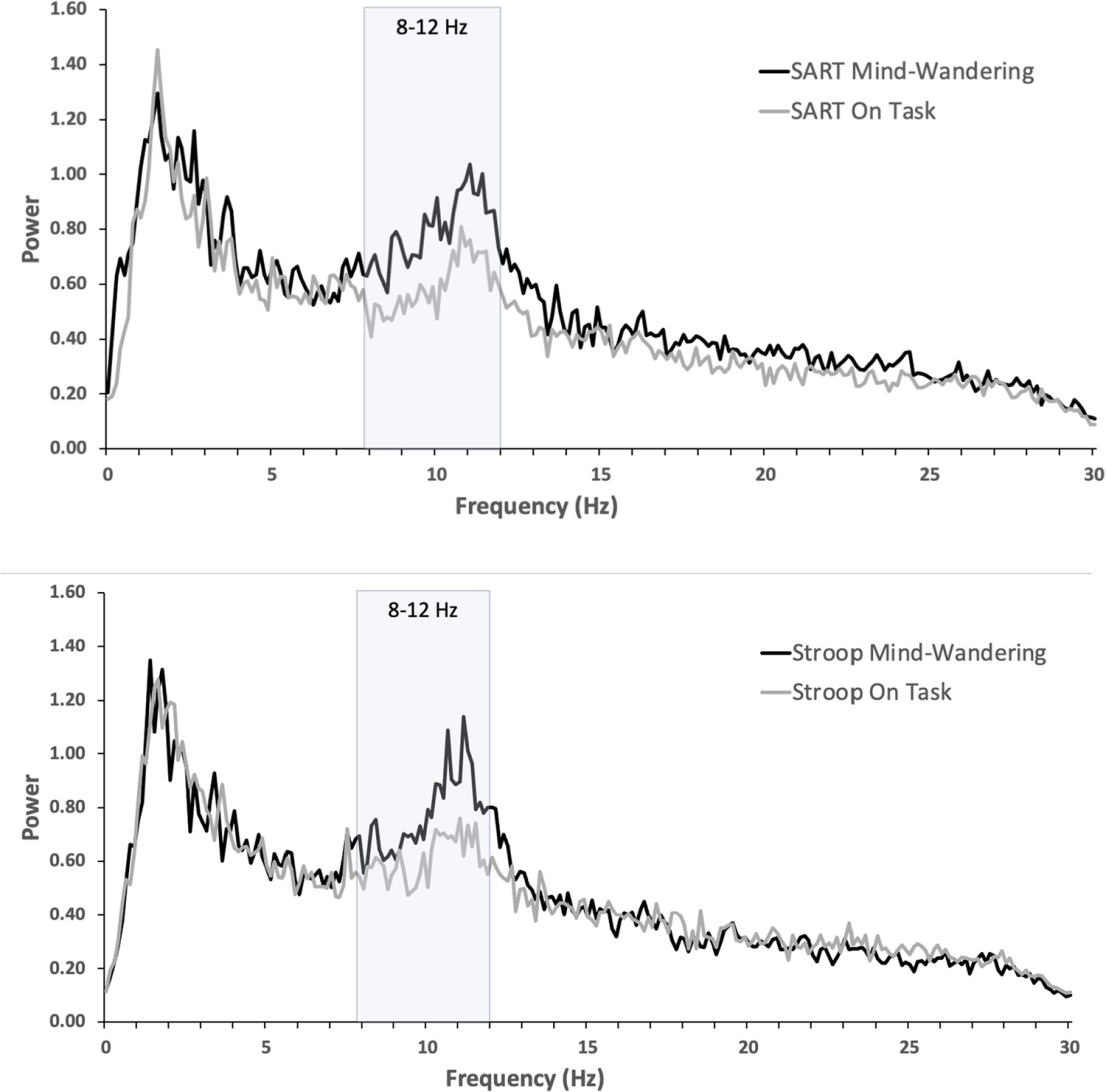
Power spectrum (Pz site) for SART and Stroop tasks, averaged separately for mind-wandering and on-task episodes

The overall goal of this set of analyses was to determine whether prior reports of increased alpha oscillations while mind-wandering could be replicated and to determine the extent to which such effects varied across task contexts. Figure 2 depicts the power spectrum for oscillations between 1 and 30 Hz, separately for the two tasks and for mind-wandering versus on-task episodes, at the Pz electrode site. Patterns in the figure suggest that frequencies in the alpha range, but not other frequencies, appear to be increased by mind-wandering.

To address these patterns statistically, alpha power values were submitted to a mixed-factorial Analysis of Variance (ANOVA), which included the repeated-measures factors Task (SART, Stroop), Probe Response (MW, OT), Region (frontal, central, parietal site), and Laterality (left, midline, right hemisphere site), and the between-subjects factor Task Order (SART first/Stroop second, Stroop first/SART second). Greenhouse-Geisser corrections were applied to *p*-values to correct for violations of sphericity in effects involving electrode site (Region, Laterality).

**Fig. 3:**
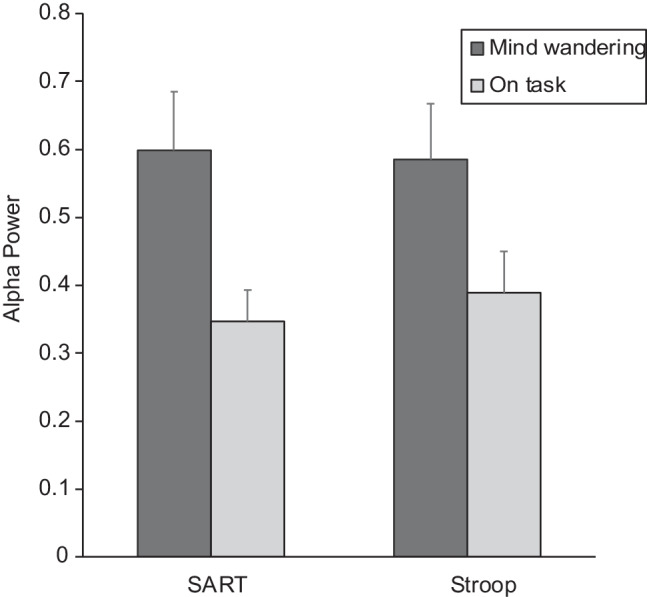
Alpha power (μV^2^) during mind-wandering versus on-task epochs, separately for the two tasks. *Note*: Error bars are ± standard error of the mean

Key results replicated the prior finding of greater alpha power during mind-wandering and found that it was consistent across the two tasks. The main effect of Probe Response (*F*(1, 35) = 13.59, *p* < .001) was due to higher alpha for the epochs before a MW self-report (*M* = 0.591 μV^2^, standard error of the mean [*SEM*] = 0.075) compared with an OT self-report (*M* = 0.368 μV^2^, *SEM* = 0.043). This effect did not further interact with Task (*F* < 1), indicating that the alpha increase associated with mind-wandering was similar across both the SART and Stroop tasks. There was no main effect of Task (*F* < 1), indicating no evidence of overall alpha differences between the tasks. These findings are summarized in Fig. 3.

Several effects indicated regional differences in alpha power. The main effect of Laterality (*F*(2, 70) = 19.05, *p* < .001) was due to highest alpha at midline sites (*M* = 0.538 μV^2^) followed by right (*M* = 0.471 μV^2^) and left (*M* = 0.429 μV^2^) hemisphere sites (all sites differ, Tukey’s HSD, *p*s < .01). The interaction of Laterality and Region (*F*(4, 140) = 5.45, *p* = .003) was due to the fact that the parietal region showed laterality effects in alpha to a greater extent than frontal and central regions (Table 4). Finally, the interaction of Laterality and Probe Response (*F*(2, 70) = 6.52, *p* = .003) reflected that although MW-OT differences were significant at each level of Laterality (Tukey’s HSD, *p*s < .02), the MW-OT differences were largest at midline sites (Table 5).

**Table 4:** Mean (SEM) alpha power (μV^2^) at nine scalp sites

	Left	Midline	Right
Frontal	0.451 (0.055)	0.500 (0.061)	0.478 (0.061)
Central	0.411 (0.046)	0.532 (0.065)	0.438 (0.050)
Parietal	0.425 (0.052)	0.583 (0.080)	0.497 (0.064)

**Table 5:** Mean (SEM) alpha power (μV^2^) across levels of laterality for mind-wandering and on-task epochs

	Mind wandering	On task	Difference (MW – OT)
Left	0.520 (0.065)	0.338 (0.039)	0.182
Midline	0.668 (0.089)	0.409 (0.051)	0.259
Right	0.586 (0.074)	0.356 (0.042)	0.230

Finally, three unanticipated interactions involved the Task Order factor, indicating that scalp distribution of alpha differed depending on which task was first (Task Order x Region, *F*(2,70) = 4.58, *p* = .036; Task Order x Region x Laterality, *F*(4, 140) = 3.70, *p* = .019; Task x Task Order x Region x Laterality, *F*(4, 140) = 3.30, *p* = .045). Means for the four-way interaction are listed in Table 6. The pattern of these interactions was not further statistically analyzed, because the interactions did not involve the mind-wandering factor, higher-level interactions are difficult to decompose, and the *p*-values, especially for the four-way interaction, were not highly robust. Nevertheless, the pattern suggests that for the SART task, alpha was more frontally distributed when the SART was first versus second, whereas for the Stroop task, alpha was more frontally distributed when the Stroop was second versus first. No other main effects or interactions in the ANOVA were significant (see Supplementary Table 1).

**Table 6:** Mean alpha power (μV^2^) across nine sites for the SART and Stroop task administered first or second in the testing session

	Task position in session		
Site	First	Second	Difference
*SART*			
F3	0.532	0.325	0.207
Fz	0.584	0.368	0.216
F4	0.575	0.344	0.231
C3	0.494	0.316	0.178
Cz	0.607	0.432	0.175
C4	0.521	0.360	0.161
P3	0.458	0.426	0.032
Pz	0.630	0.554	0.076
P4	0.480	0.494	−0.014
*Stroop*			
F3	0.396	0.552	−0.156
Fz	0.428	0.620	−0.192
F4	0.390	0.605	−0.215
C3	0.373	0.461	−0.088
Cz	0.490	0.600	−0.110
C4	0.385	0.486	−0.101
P3	0.393	0.422	−0.029
Pz	0.641	0.506	0.135
P4	0.617	0.398	0.219

Furthermore, when individual differences in the degree of deliberate mind-wandering were added to the ANOVA as a covariate, no effects involving this variable were significant,

indicating that individual differences in deliberate mind-wandering did not predict the modulation of alpha across task conditions. Summarizing the key findings from the alpha oscillations, alpha power values were higher during mind-wandering episodes than when on-task, for both the SART and the Stroop.

### ERP measures

**Fig. 4:**
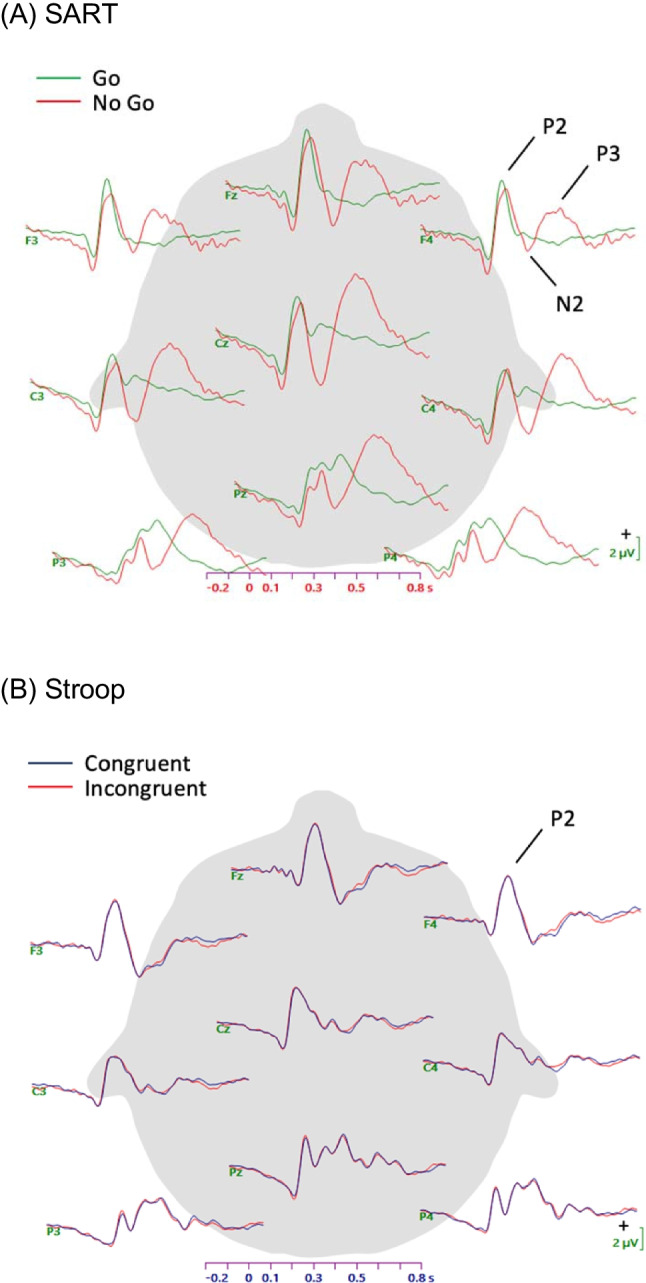
Grand-average ERP waveforms for SART and Stroop tasks (all trials, n = 48)

Grand-averages depicting the ERP waveforms (including all trials) are illustrated in Fig. 4 for both the SART and Stroop tasks. These grand averages include all participants with usable EEG data (*n* = 48). Visual inspection of the SART waveforms suggests the expected increase in N2 and P3 amplitudes for no-go trials compared with go trials. Visual inspection of the Stroop waveforms indicates highly similar ERPs, with typical components, for the congruent and incongruent trials. These waveforms demonstrate that the tasks as a whole produced expected ERP components. Because the P2 component was the most clearly evident component across both tasks, this component was selected for analysis to compare between tasks and mind-wandering/on-task episodes.

**Fig. 5:**
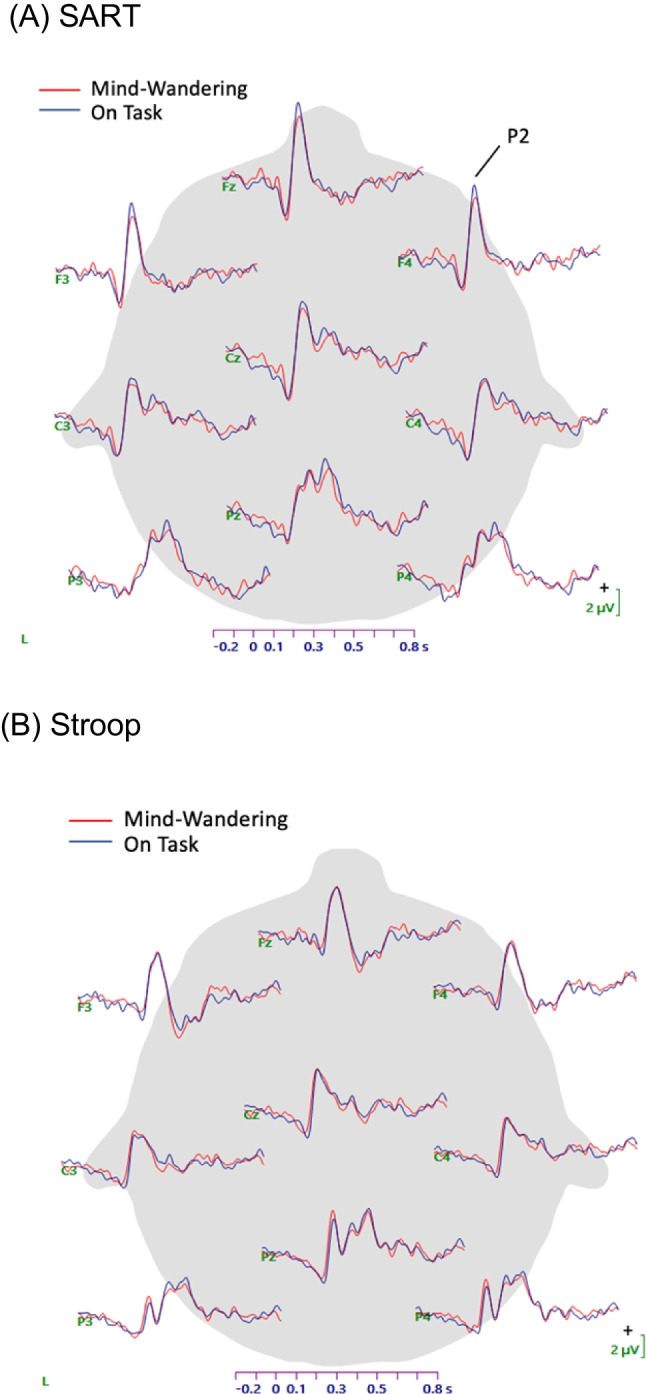
ERP waveforms for the SART and Stroop tasks for trials that occurred during mind-wandering or on-task episodes (n = 37)

To examine the effect of mind-wandering during the SART and Stroop, we calculated waveforms that included five trials before each experience-sampling probe, sorted according to whether the participant responded to the probe indicating that they were mind-wandering or on-task. Only go trials were included for the SART, because the infrequency of no-go trials together with the infrequency of probes could lead to unevenness of no-go trial contribution across participants. Figure 5 displays the resulting waveforms for both the SART and the Stroop. P2 peak amplitudes were extracted as described in the method section. For this analysis, only participants with at least three instances of mind-wandering and three instances of on-task self-report for each task were included (*n* = 37).

The main questions of interest are whether P2 amplitudes would be reduced while mind-wandering and whether this expected reduction would be consistent across the two tasks. To address this, P2 peak amplitudes were submitted to an ANOVA with Task, Probe Response, Region, and Laterality as repeated-measures factors and Task Order as a between-subjects factor.

**Fig. 6:**
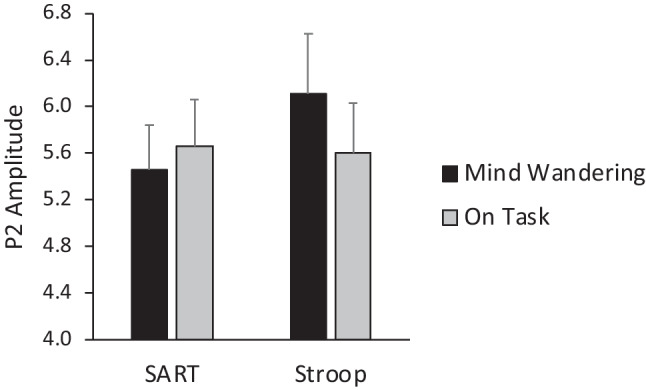
Amplitude of the P2 peak (in μV) was differently affected by mind-wandering for the SART versus the Stroop task. *Note*: Error bars are ± SEM

The main effect of Probe Response was not significant (*p* > .40), whereas the Task x Probe Response interaction was significant (*F*(1, 35) = 5.46, *p* = .025). The means for the interaction are displayed in Fig. 6. The pattern of the interaction indicates that the expected reduction of the P2 amplitude while mind-wandering was reversed for the Stroop task. Post-hoc comparisons found that the P2 during mind-wandering was greater for the Stroop than the SART (*p* = .051, uncorrected), while P2 amplitudes during on-task episodes did not differ between tasks (*p* > .80). Moreover, the difference between mind-wandering and on-task P2 amplitudes approached significance for the Stroop task (*p* = .053) but not the SART (*p* > .40).

**Fig. 7:**
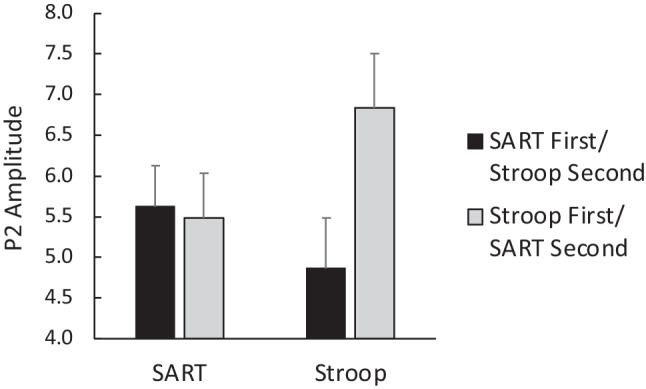
P2 amplitudes (in μV) were enhanced for the Stroop task when it was completed first versus second. *Note*: Error bars are ± SEM

A second task-related effect was the Task x Task Order interaction (*F*(1, 35) = 12.30, *p* = .001). Means for the interaction are presented in Fig. 7 and indicate that P2 amplitudes during the Stroop task were enhanced when the Stroop task was first rather than second (post-hoc comparison, *p* = .038), whereas P2 amplitudes for the SART task were not affected by task order (*p* > .80).

Additional effects for the P2 analyses reflected the scalp distribution of the P2. The main effect of Region (*F*(2, 70) = 12.83, *p* < .001) was due to highest P2 at frontal sites (*M* = 6.55 μV) followed by central sites (*M* = 5.59 μV) and smallest amplitudes at parietal sites (*M* = 4.99 μV). The main effect of Laterality (*F*(2, 70) = 10.33, *p* < .001) was due to highest P2 at midline sites (*M* = 5.97 μV) compared with left hemisphere (*M* = 5.60 μV) and right hemisphere (*M* = 5.55 μV). No other effects in the ANOVA were significant (see Supplementary Table 2). Finally, the individual differences variable tapping deliberate mind-wandering was entered into the analysis as a covariate and produced no significant effects.

## Discussion

The main goal of the study was to examine how neural correlates of mind-wandering vary according to the demands of the external task. Consistency in the association between mind-wandering self-report and neural markers, such as EEG/ERP indices, can provide evidence of generalizability of the phenomena across different cognitive tasks. Conversely, where different patterns of association between mind-wandering and neural measures emerge across different tasks, the evidence may advance our understanding of how participants modulate mind-wandering across differing contexts. Overall, results from the present study provide some evidence of consistency in neural markers of mind-wandering as well as evidence of task- or context-specific modulation. Results are interpreted in relation to the context-regulation hypothesis of mind-wandering, the perceptual decoupling theory, and the theoretical distinction between deliberate and spontaneous mind-wandering. Generally, results support the concept of context regulation, offer mixed support for the perceptual decoupling idea, and do not provide strong support for the deliberate-spontaneous distinction.

Self-report and performance data replicate some prior findings and support the idea that the frequency of mind-wandering varies systematically across task contexts. Participants reported mind-wandering approximately half of the time, on average, consistent with previous findings of mind-wandering rates between 30% and 50% (Kane et al., 2017; Killingsworth & Gilbert, 2010; Seli et al., 2016). Moreover, mind-wandering was more frequently reported during the less-demanding SART task than the more-demanding Stroop task, and it was more frequent during the second of the two tasks compared with the first of the two tasks, which were presented in counterbalanced order. These results support the context regulation idea by demonstrating how both the task demands and the session time elapsed can affect the self-reported tendency for the mind to drift off-task. Moreover, the negative correlations between individual differences in mind-wandering and performance on both tasks reveals that even as participants adjust mind-wandering frequency depending on the task demands in ways that may seem adaptive, there is nevertheless a performance decrement among those who report more mind-wandering overall (Mrazek et al., 2012a, b; Randall et al., 2014).

Results examining the relationship between mind-wandering and EEG alpha oscillations both replicated and extended previous findings. Overall, the tendency for mind-wandering episodes to be associated with increased alpha oscillations was confirmed, supporting previous studies (Arnau et al., 2020; Compton et al., 2019; da Silva et al., 2022) and demonstrating generalizability of the finding across the SART and Stroop tasks. However, the alpha oscillations did not directly align with the variation in mind-wandering self-reports across tasks. Alpha oscillations were not higher overall for the easier SART or for the second task compared with the first task, as might be predicted if they directly tracked the context modulation of participant’s self-reports of mind-wandering. When participants reported mind-wandering, alpha power was elevated, regardless of the specific task or session position of the task. Thus, while self-report data support the context regulation of mind-wandering frequency, the oscillatory EEG suggest that alpha power as a correlate of mind-wandering is consistent across task contexts.

The perceptual decoupling theory posits that the brain is less responsive to external stimuli while mind-wandering and has been supported in past research by reduced ERP responses to stimuli during mind-wandering episodes (Handy & Kam, 2015; Kam et al., 2022; Smallwood et al., 2008). The present study was designed to replicate that finding and to determine whether it was modulated by task difficulty. Previous studies about mind-wandering have used a range of ERP components, including early sensory-perceptual components, such as the P2 as well as later-occurring components, such as the P3, to test the perceptual decoupling hypothesis (Kam et al., 2022). We selected the P2 component of the waveform for statistical analysis, because it was the most salient ERP component across both SART and Stroop tasks in our grand-average waveforms (Fig. 4). Although the P3 component was visibly evident on the infrequent No-Go trials in the SART, the absence of the P3 on the frequent Go trials and its limited presence in the Stroop trials disqualified it from consideration in our ERP mind-wandering analysis.

Results from our analysis of the P2 component were unexpectedly inconsistent with predictions from the perceptual decoupling hypothesis. While the SART showed a pattern of means in the expected direction, namely slightly (but not significantly) lower P2 amplitudes while mind-wandering than when on-task, the Stroop task unexpectedly produced the opposite pattern. That is, mind-wandering during the Stroop task was associated with an enhanced, rather than reduced, P2 compared with on-task thought.

In a general sense, the P2 results support the broad conclusion that task context modulates the neural markers associated with mind-wandering. We predicted that perceptual decoupling would be lessened during a harder task, as participants might allocate more attention to the stimuli for a harder task, compared with an easier task in which perception could be more decoupled while still maintaining task performance. However, it is challenging to interpret why the P2 amplitude pattern reversed with the more difficult Stroop task, such that the peak was higher while mind-wandering compared to on-task episodes. Existing theories do not easily accommodate this finding, which directly contradicts the perceptual decoupling theory. Notably, most previous studies of perceptual decoupling have examined mind-wandering during easy tasks, such as the SART or simple oddball tasks (Kam et al., 2022). One possibility that future studies might explore is that the mechanism of perceptual decoupling only applies to simple tasks that can be completed with minimal external attention; in contrast, in more difficult tasks (such as the Stroop task), processing of external stimuli must be sustained and possibly even enhanced while mind-wandering to maintain adequate task performance. This interpretation remains speculative, given the unexpected finding, which should be replicated in other datasets.

Finally, evidence from the present study did not strongly support the relevance of the deliberate-spontaneous distinction to understanding neural correlates of mind-wandering. Participants’ self-reports of deliberate (versus spontaneous) mind-wandering were consistent across measurements (i.e., post-task estimates for both tasks as well as general retrospective estimate), implying some stable individual difference. Nevertheless, the composite index of deliberate mind-wandering did not predict task performance or neural markers of mind-wandering. Self-reported deliberate mind-wandering also did not correlate significantly with an independent measure of executive functioning, the BRIEF inventory. This null result is inconsistent with the idea that deliberate mind-wandering reflects adaptive cognitive control. Because the deliberate-spontaneous dimension of mind-wandering is supported by other research using behavioral and self-report methods (Seli et al., 2015, 2016, 2018, 2019), it may still be a useful dimension in other areas of study. In the present study, the reliance on retrospective report to measure deliberate mind-wandering, along with the more general challenge inherent in self-evaluating the deliberateness of one’s mind-wandering, may have limited the validity of the measure. In any case, the present results do not offer support for the relationship of individual differences in deliberate mind-wandering with the particular EEG/ERP markers that we investigated.

While the present results expand understanding of how neural correlates of mind-wandering may be affected by task contexts, the study has several limitations. First, the SART and the Stroop task differ along several different dimensions: for example, one is a task of sustained attention and the other a task of selective attention; one involves a more complex response mapping than the other; one involves color stimuli and the other does not; one involves numbers and the other involves words. Thus, while the tasks certainly differ in difficulty level, any number of specific task attributes may account for that difference, and the present study does not attempt to tease apart those attributes. Future studies might systematically manipulate one aspect of task difficulty (e.g., working memory load, or number of response options in the Stroop) to confirm and further elucidate the effect of task difficulty on mind-wandering.

A second limitation is that each task contained only 15 experience-sampling probes because of the limited task length and the desirability of having probes that were fairly reasonably spaced apart (Robison et al., 2019; Seli et al., 2013). As a consequence, a relatively small number of trials contributes to the ERP and EEG comparisons between on-task versus mind-wandering episodes, which were identified by responses to those probes. Because mind-wandering frequency differs across participants, some participants reported mind-wandering (or being on task) in response to only one or two of the 15 probes. These participants were excluded from key analyses due to insufficient numbers of episodes. Accordingly, instances of null results may be viewed with more skepticism due to possible lack of statistical power. Finally, the sample of participants itself is limited in age range and other demographic factors that could potentially influence aspects of attentional control.

Despite the limitations of the study, these results contribute to understanding of the neural mechanisms of mind-wandering by demonstrating how they are modulated by task factors. We confirmed that participants reported more mind-wandering during an easier task and during the second of two tasks in the session. Furthermore, while oscillatory EEG correlates of mind-wandering remained consistent across task contexts, ERP correlates of mind-wandering were influenced by task. Analysis of the P2 ERP component indicated that the theory of perceptual decoupling during mind-wandering may not account well for stimulus processing during more difficult external tasks. Thus, the present EEG data add nuance to the relatively simple neural associations put forth previously and suggest avenues for additional research.

### Supplementary Information

Below is the link to the electronic supplementary material.

Supplementary file1 (DOCX 25 KB)

Supplementary file2 (DOCX 25 KB)

## Data Availability

Raw data, task scripts, and data processing scripts are available at https://osf.io/6gtns/?view_only=4e19c65bb3604ff8acd463833e75d454
