# Supplementary material for: Effects of task context on EEG correlates of mind-wandering
Source: Cogn Affect Behav Neurosci. 2023 Nov 29;24(1):72–86. doi: 10.3758/s13415-023-01138-9 (PMC10827903; doi:10.3758/s13415-023-01138-9)
Supplement: Supplementary file 1 — Supplementary file1 (DOCX 25 KB) [file 13415_2023_1138_MOESM1_ESM.docx]

Supplementary Table 1. Full ANOVA results for ANOVA on alpha power

| Within Subjects Effects | | | | | | | | | | | | | |
| --- | --- | --- | --- | --- | --- | --- | --- | --- | --- | --- | --- | --- | --- |
|  | | **Sphericity Correction** | | **Sum of Squares** | | **df** | | **Mean Square** | | **F** | | **p** | |
| Task |  | None |  | 0.07038 |  | 1 |  | 0.07038 |  | 0.08265 |  | 0.775 |  |
|  |  | Greenhouse-Geisser |  | 0.07038 |  | 1.00 |  | 0.07038 |  | 0.08265 |  | 0.775 |  |
| Task ✻ FirstTask |  | None |  | 0.54165 |  | 1 |  | 0.54165 |  | 0.63605 |  | 0.431 |  |
|  |  | Greenhouse-Geisser |  | 0.54165 |  | 1.00 |  | 0.54165 |  | 0.63605 |  | 0.431 |  |
| Residual |  | None |  | 29.80505 |  | 35 |  | 0.85157 |  |  |  |  |  |
|  |  | Greenhouse-Geisser |  | 29.80505 |  | 35.00 |  | 0.85157 |  |  |  |  |  |
| ProbeResponse |  | None |  | 16.61041 |  | 1 |  | 16.61041 |  | 13.59485 |  | < .001 |  |
|  |  | Greenhouse-Geisser |  | 16.61041 |  | 1.00 |  | 16.61041 |  | 13.59485 |  | < .001 |  |
| ProbeResponse ✻ FirstTask |  | None |  | 0.00890 |  | 1 |  | 0.00890 |  | 0.00728 |  | 0.932 |  |
|  |  | Greenhouse-Geisser |  | 0.00890 |  | 1.00 |  | 0.00890 |  | 0.00728 |  | 0.932 |  |
| Residual |  | None |  | 42.76357 |  | 35 |  | 1.22182 |  |  |  |  |  |
|  |  | Greenhouse-Geisser |  | 42.76357 |  | 35.00 |  | 1.22182 |  |  |  |  |  |
| Region |  | None |  | 0.37862 |  | 2 |  | 0.18931 |  | 0.50275 |  | 0.607 |  |
|  |  | Greenhouse-Geisser |  | 0.37862 |  | 1.09 |  | 0.34798 |  | 0.50275 |  | 0.498 |  |
| Region ✻ FirstTask |  | None |  | 3.44791 |  | 2 |  | 1.72396 |  | 4.57825 |  | 0.014 |  |
|  |  | Greenhouse-Geisser |  | 3.44791 |  | 1.09 |  | 3.16885 |  | 4.57825 |  | 0.036 |  |
| Residual |  | None |  | 26.35875 |  | 70 |  | 0.37655 |  |  |  |  |  |
|  |  | Greenhouse-Geisser |  | 26.35875 |  | 38.08 |  | 0.69215 |  |  |  |  |  |
| Laterality |  | None |  | 2.69946 |  | 2 |  | 1.34973 |  | 19.04548 |  | < .001 |  |
|  |  | Greenhouse-Geisser |  | 2.69946 |  | 1.63 |  | 1.65564 |  | 19.04548 |  | < .001 |  |
| Laterality ✻ FirstTask |  | None |  | 0.07833 |  | 2 |  | 0.03917 |  | 0.55266 |  | 0.578 |  |
|  |  | Greenhouse-Geisser |  | 0.07833 |  | 1.63 |  | 0.04804 |  | 0.55266 |  | 0.543 |  |
| Residual |  | None |  | 4.96082 |  | 70 |  | 0.07087 |  |  |  |  |  |
|  |  | Greenhouse-Geisser |  | 4.96082 |  | 57.07 |  | 0.08693 |  |  |  |  |  |
| Task ✻ ProbeResponse |  | None |  | 0.25595 |  | 1 |  | 0.25595 |  | 0.32597 |  | 0.572 |  |
|  |  | Greenhouse-Geisser |  | 0.25595 |  | 1.00 |  | 0.25595 |  | 0.32597 |  | 0.572 |  |
| Task ✻ ProbeResponse ✻ FirstTask |  | None |  | 0.10178 |  | 1 |  | 0.10178 |  | 0.12962 |  | 0.721 |  |
|  |  | Greenhouse-Geisser |  | 0.10178 |  | 1.00 |  | 0.10178 |  | 0.12962 |  | 0.721 |  |
| Residual |  | None |  | 27.48274 |  | 35 |  | 0.78522 |  |  |  |  |  |
|  |  | Greenhouse-Geisser |  | 27.48274 |  | 35.00 |  | 0.78522 |  |  |  |  |  |
| Task ✻ Region |  | None |  | 0.16843 |  | 2 |  | 0.08422 |  | 2.18005 |  | 0.121 |  |
|  |  | Greenhouse-Geisser |  | 0.16843 |  | 1.58 |  | 0.10678 |  | 2.18005 |  | 0.133 |  |
| Task ✻ Region ✻ FirstTask |  | None |  | 0.17257 |  | 2 |  | 0.08629 |  | 2.23364 |  | 0.115 |  |
|  |  | Greenhouse-Geisser |  | 0.17257 |  | 1.58 |  | 0.10940 |  | 2.23364 |  | 0.127 |  |
| Residual |  | None |  | 2.70411 |  | 70 |  | 0.03863 |  |  |  |  |  |
|  |  | Greenhouse-Geisser |  | 2.70411 |  | 55.21 |  | 0.04898 |  |  |  |  |  |
| ProbeResponse ✻ Region |  | None |  | 0.23410 |  | 2 |  | 0.11705 |  | 1.22514 |  | 0.300 |  |
|  |  | Greenhouse-Geisser |  | 0.23410 |  | 1.27 |  | 0.18499 |  | 1.22514 |  | 0.286 |  |
| ProbeResponse ✻ Region ✻ FirstTask |  | None |  | 0.17331 |  | 2 |  | 0.08666 |  | 0.90701 |  | 0.408 |  |
|  |  | Greenhouse-Geisser |  | 0.17331 |  | 1.27 |  | 0.13696 |  | 0.90701 |  | 0.369 |  |
| Residual |  | None |  | 6.68781 |  | 70 |  | 0.09554 |  |  |  |  |  |
|  |  | Greenhouse-Geisser |  | 6.68781 |  | 44.29 |  | 0.15100 |  |  |  |  |  |
| Task ✻ Laterality |  | None |  | 0.00804 |  | 2 |  | 0.00402 |  | 0.11209 |  | 0.894 |  |
|  |  | Greenhouse-Geisser |  | 0.00804 |  | 1.60 |  | 0.00504 |  | 0.11209 |  | 0.850 |  |
| Task ✻ Laterality ✻ FirstTask |  | None |  | 0.04385 |  | 2 |  | 0.02193 |  | 0.61109 |  | 0.546 |  |
|  |  | Greenhouse-Geisser |  | 0.04385 |  | 1.60 |  | 0.02747 |  | 0.61109 |  | 0.511 |  |
| Residual |  | None |  | 2.51167 |  | 70 |  | 0.03588 |  |  |  |  |  |
|  |  | Greenhouse-Geisser |  | 2.51167 |  | 55.88 |  | 0.04495 |  |  |  |  |  |
| ProbeResponse ✻ Laterality |  | None |  | 0.32656 |  | 2 |  | 0.16328 |  | 6.52030 |  | 0.003 |  |
|  |  | Greenhouse-Geisser |  | 0.32656 |  | 1.92 |  | 0.16981 |  | 6.52030 |  | 0.003 |  |
| ProbeResponse ✻ Laterality ✻ FirstTask |  | None |  | 0.05849 |  | 2 |  | 0.02925 |  | 1.16789 |  | 0.317 |  |
|  |  | Greenhouse-Geisser |  | 0.05849 |  | 1.92 |  | 0.03042 |  | 1.16789 |  | 0.316 |  |
| Residual |  | None |  | 1.75290 |  | 70 |  | 0.02504 |  |  |  |  |  |
|  |  | Greenhouse-Geisser |  | 1.75290 |  | 67.31 |  | 0.02604 |  |  |  |  |  |
| Region ✻ Laterality |  | None |  | 0.53120 |  | 4 |  | 0.13280 |  | 5.45409 |  | < .001 |  |
|  |  | Greenhouse-Geisser |  | 0.53120 |  | 2.56 |  | 0.20713 |  | 5.45409 |  | 0.003 |  |
| Region ✻ Laterality ✻ FirstTask |  | None |  | 0.36079 |  | 4 |  | 0.09020 |  | 3.70440 |  | 0.007 |  |
|  |  | Greenhouse-Geisser |  | 0.36079 |  | 2.56 |  | 0.14068 |  | 3.70440 |  | 0.019 |  |
| Residual |  | None |  | 3.40880 |  | 140 |  | 0.02435 |  |  |  |  |  |
|  |  | Greenhouse-Geisser |  | 3.40880 |  | 89.76 |  | 0.03798 |  |  |  |  |  |
| Task ✻ ProbeResponse ✻ Region |  | None |  | 0.01474 |  | 2 |  | 0.00737 |  | 0.17860 |  | 0.837 |  |
|  |  | Greenhouse-Geisser |  | 0.01474 |  | 1.54 |  | 0.00956 |  | 0.17860 |  | 0.780 |  |
| Task ✻ ProbeResponse ✻ Region ✻ FirstTask |  | None |  | 0.09849 |  | 2 |  | 0.04924 |  | 1.19370 |  | 0.309 |  |
|  |  | Greenhouse-Geisser |  | 0.09849 |  | 1.54 |  | 0.06387 |  | 1.19370 |  | 0.301 |  |
| Residual |  | None |  | 2.88769 |  | 70 |  | 0.04125 |  |  |  |  |  |
|  |  | Greenhouse-Geisser |  | 2.88769 |  | 53.97 |  | 0.05351 |  |  |  |  |  |
| Task ✻ ProbeResponse ✻ Laterality |  | None |  | 0.03315 |  | 2 |  | 0.01658 |  | 0.70631 |  | 0.497 |  |
|  |  | Greenhouse-Geisser |  | 0.03315 |  | 1.94 |  | 0.01706 |  | 0.70631 |  | 0.493 |  |
| Task ✻ ProbeResponse ✻ Laterality ✻ FirstTask |  | None |  | 0.02519 |  | 2 |  | 0.01259 |  | 0.53655 |  | 0.587 |  |
|  |  | Greenhouse-Geisser |  | 0.02519 |  | 1.94 |  | 0.01296 |  | 0.53655 |  | 0.582 |  |
| Residual |  | None |  | 1.64285 |  | 70 |  | 0.02347 |  |  |  |  |  |
|  |  | Greenhouse-Geisser |  | 1.64285 |  | 68.01 |  | 0.02416 |  |  |  |  |  |
| Task ✻ Region ✻ Laterality |  | None |  | 0.07081 |  | 4 |  | 0.01770 |  | 1.02641 |  | 0.396 |  |
|  |  | Greenhouse-Geisser |  | 0.07081 |  | 1.91 |  | 0.03708 |  | 1.02641 |  | 0.361 |  |
| Task ✻ Region ✻ Laterality ✻ FirstTask |  | None |  | 0.22790 |  | 4 |  | 0.05697 |  | 3.30348 |  | 0.013 |  |
|  |  | Greenhouse-Geisser |  | 0.22790 |  | 1.91 |  | 0.11934 |  | 3.30348 |  | 0.045 |  |
| Residual |  | None |  | 2.41454 |  | 140 |  | 0.01725 |  |  |  |  |  |
|  |  | Greenhouse-Geisser |  | 2.41454 |  | 66.84 |  | 0.03613 |  |  |  |  |  |
| ProbeResponse ✻ Region ✻ Laterality |  | None |  | 0.08861 |  | 4 |  | 0.02215 |  | 1.58513 |  | 0.182 |  |
|  |  | Greenhouse-Geisser |  | 0.08861 |  | 1.90 |  | 0.04674 |  | 1.58513 |  | 0.213 |  |
| ProbeResponse ✻ Region ✻ Laterality ✻ FirstTask |  | None |  | 0.12036 |  | 4 |  | 0.03009 |  | 2.15305 |  | 0.077 |  |
|  |  | Greenhouse-Geisser |  | 0.12036 |  | 1.90 |  | 0.06348 |  | 2.15305 |  | 0.127 |  |
| Residual |  | None |  | 1.95663 |  | 140 |  | 0.01398 |  |  |  |  |  |
|  |  | Greenhouse-Geisser |  | 1.95663 |  | 66.36 |  | 0.02948 |  |  |  |  |  |
| Task ✻ ProbeResponse ✻ Region ✻ Laterality |  | None |  | 0.01216 |  | 4 |  | 0.00304 |  | 0.22193 |  | 0.926 |  |
|  |  | Greenhouse-Geisser |  | 0.01216 |  | 1.76 |  | 0.00691 |  | 0.22193 |  | 0.773 |  |
| Task ✻ ProbeResponse ✻ Region ✻ Laterality ✻ FirstTask |  | None |  | 0.08653 |  | 4 |  | 0.02163 |  | 1.57961 |  | 0.183 |  |
|  |  | Greenhouse-Geisser |  | 0.08653 |  | 1.76 |  | 0.04921 |  | 1.57961 |  | 0.216 |  |
| Residual |  | None |  | 1.91734 |  | 140 |  | 0.01370 |  |  |  |  |  |
|  |  | Greenhouse-Geisser |  | 1.91734 |  | 61.55 |  | 0.03115 |  |  |  |  |  |
| Note. Type 3 Sums of Squares | | | | | | | | | | | | | |
|  | | | | | | | | | | | | | |
