# Supplementary material for: Effects of task context on EEG correlates of mind-wandering
Source: Cogn Affect Behav Neurosci. 2023 Nov 29;24(1):72–86. doi: 10.3758/s13415-023-01138-9 (PMC10827903; doi:10.3758/s13415-023-01138-9)
Supplement: Supplementary file 2 — Supplementary file2 (DOCX 25 KB) [file 13415_2023_1138_MOESM2_ESM.docx]

Supplementary Table 2. Full ANOVA results from analyses of P2 peaks.

| Within Subjects Effects | | | | | | | | | | | | | |
| --- | --- | --- | --- | --- | --- | --- | --- | --- | --- | --- | --- | --- | --- |
|  | | **Sphericity Correction** | | **Sum of Squares** | | **df** | | **Mean Square** | | **F** | | **p** | |
| Task |  | None |  | 28.637 |  | 1 |  | 28.637 |  | 0.964 |  | 0.333 |  |
|  |  | Greenhouse-Geisser |  | 28.637 |  | 1.00 |  | 28.637 |  | 0.964 |  | 0.333 |  |
| Task ✻ FirstTask |  | None |  | 365.467 |  | 1 |  | 365.467 |  | 12.304 |  | 0.001 |  |
|  |  | Greenhouse-Geisser |  | 365.467 |  | 1.00 |  | 365.467 |  | 12.304 |  | 0.001 |  |
| Residual |  | None |  | 1039.623 |  | 35 |  | 29.704 |  |  |  |  |  |
|  |  | Greenhouse-Geisser |  | 1039.623 |  | 35.00 |  | 29.704 |  |  |  |  |  |
| ProbeResponse |  | None |  | 8.315 |  | 1 |  | 8.315 |  | 0.616 |  | 0.438 |  |
|  |  | Greenhouse-Geisser |  | 8.315 |  | 1.00 |  | 8.315 |  | 0.616 |  | 0.438 |  |
| ProbeResponse ✻ FirstTask |  | None |  | 3.170 |  | 1 |  | 3.170 |  | 0.235 |  | 0.631 |  |
|  |  | Greenhouse-Geisser |  | 3.170 |  | 1.00 |  | 3.170 |  | 0.235 |  | 0.631 |  |
| Residual |  | None |  | 472.218 |  | 35 |  | 13.492 |  |  |  |  |  |
|  |  | Greenhouse-Geisser |  | 472.218 |  | 35.00 |  | 13.492 |  |  |  |  |  |
| Region |  | None |  | 546.236 |  | 2 |  | 273.118 |  | 12.831 |  | < .001 |  |
|  |  | Greenhouse-Geisser |  | 546.236 |  | 1.17 |  | 468.822 |  | 12.831 |  | < .001 |  |
| Region ✻ FirstTask |  | None |  | 103.649 |  | 2 |  | 51.825 |  | 2.435 |  | 0.095 |  |
|  |  | Greenhouse-Geisser |  | 103.649 |  | 1.17 |  | 88.960 |  | 2.435 |  | 0.122 |  |
| Residual |  | None |  | 1489.992 |  | 70 |  | 21.286 |  |  |  |  |  |
|  |  | Greenhouse-Geisser |  | 1489.992 |  | 40.78 |  | 36.538 |  |  |  |  |  |
| Laterality |  | None |  | 47.697 |  | 2 |  | 23.849 |  | 10.329 |  | < .001 |  |
|  |  | Greenhouse-Geisser |  | 47.697 |  | 1.82 |  | 26.200 |  | 10.329 |  | < .001 |  |
| Laterality ✻ FirstTask |  | None |  | 14.496 |  | 2 |  | 7.248 |  | 3.139 |  | 0.049 |  |
|  |  | Greenhouse-Geisser |  | 14.496 |  | 1.82 |  | 7.963 |  | 3.139 |  | 0.055 |  |
| Residual |  | None |  | 161.627 |  | 70 |  | 2.309 |  |  |  |  |  |
|  |  | Greenhouse-Geisser |  | 161.627 |  | 63.72 |  | 2.537 |  |  |  |  |  |
| Task ✻ ProbeResponse |  | None |  | 42.345 |  | 1 |  | 42.345 |  | 5.464 |  | 0.025 |  |
|  |  | Greenhouse-Geisser |  | 42.345 |  | 1.00 |  | 42.345 |  | 5.464 |  | 0.025 |  |
| Task ✻ ProbeResponse ✻ FirstTask |  | None |  | 6.175 |  | 1 |  | 6.175 |  | 0.797 |  | 0.378 |  |
|  |  | Greenhouse-Geisser |  | 6.175 |  | 1.00 |  | 6.175 |  | 0.797 |  | 0.378 |  |
| Residual |  | None |  | 271.264 |  | 35 |  | 7.750 |  |  |  |  |  |
|  |  | Greenhouse-Geisser |  | 271.264 |  | 35.00 |  | 7.750 |  |  |  |  |  |
| Task ✻ Region |  | None |  | 7.167 |  | 2 |  | 3.584 |  | 0.531 |  | 0.591 |  |
|  |  | Greenhouse-Geisser |  | 7.167 |  | 1.43 |  | 5.014 |  | 0.531 |  | 0.532 |  |
| Task ✻ Region ✻ FirstTask |  | None |  | 21.887 |  | 2 |  | 10.943 |  | 1.620 |  | 0.205 |  |
|  |  | Greenhouse-Geisser |  | 21.887 |  | 1.43 |  | 15.313 |  | 1.620 |  | 0.212 |  |
| Residual |  | None |  | 472.726 |  | 70 |  | 6.753 |  |  |  |  |  |
|  |  | Greenhouse-Geisser |  | 472.726 |  | 50.03 |  | 9.450 |  |  |  |  |  |
| ProbeResponse ✻ Region |  | None |  | 6.374 |  | 2 |  | 3.187 |  | 1.222 |  | 0.301 |  |
|  |  | Greenhouse-Geisser |  | 6.374 |  | 1.44 |  | 4.417 |  | 1.222 |  | 0.292 |  |
| ProbeResponse ✻ Region ✻ FirstTask |  | None |  | 7.419 |  | 2 |  | 3.709 |  | 1.423 |  | 0.248 |  |
|  |  | Greenhouse-Geisser |  | 7.419 |  | 1.44 |  | 5.140 |  | 1.423 |  | 0.248 |  |
| Residual |  | None |  | 182.518 |  | 70 |  | 2.607 |  |  |  |  |  |
|  |  | Greenhouse-Geisser |  | 182.518 |  | 50.51 |  | 3.613 |  |  |  |  |  |
| Task ✻ Laterality |  | None |  | 1.253 |  | 2 |  | 0.627 |  | 0.507 |  | 0.604 |  |
|  |  | Greenhouse-Geisser |  | 1.253 |  | 1.88 |  | 0.667 |  | 0.507 |  | 0.593 |  |
| Task ✻ Laterality ✻ FirstTask |  | None |  | 5.296 |  | 2 |  | 2.648 |  | 2.143 |  | 0.125 |  |
|  |  | Greenhouse-Geisser |  | 5.296 |  | 1.88 |  | 2.818 |  | 2.143 |  | 0.128 |  |
| Residual |  | None |  | 86.505 |  | 70 |  | 1.236 |  |  |  |  |  |
|  |  | Greenhouse-Geisser |  | 86.505 |  | 65.77 |  | 1.315 |  |  |  |  |  |
| ProbeResponse ✻ Laterality |  | None |  | 0.852 |  | 2 |  | 0.426 |  | 0.403 |  | 0.670 |  |
|  |  | Greenhouse-Geisser |  | 0.852 |  | 1.53 |  | 0.558 |  | 0.403 |  | 0.615 |  |
| ProbeResponse ✻ Laterality ✻ FirstTask |  | None |  | 1.419 |  | 2 |  | 0.709 |  | 0.672 |  | 0.514 |  |
|  |  | Greenhouse-Geisser |  | 1.419 |  | 1.53 |  | 0.929 |  | 0.672 |  | 0.477 |  |
| Residual |  | None |  | 73.933 |  | 70 |  | 1.056 |  |  |  |  |  |
|  |  | Greenhouse-Geisser |  | 73.933 |  | 53.45 |  | 1.383 |  |  |  |  |  |
| Region ✻ Laterality |  | None |  | 7.260 |  | 4 |  | 1.815 |  | 1.456 |  | 0.219 |  |
|  |  | Greenhouse-Geisser |  | 7.260 |  | 3.35 |  | 2.170 |  | 1.456 |  | 0.227 |  |
| Region ✻ Laterality ✻ FirstTask |  | None |  | 20.007 |  | 4 |  | 5.002 |  | 4.013 |  | 0.004 |  |
|  |  | Greenhouse-Geisser |  | 20.007 |  | 3.35 |  | 5.979 |  | 4.013 |  | 0.007 |  |
| Residual |  | None |  | 174.486 |  | 140 |  | 1.246 |  |  |  |  |  |
|  |  | Greenhouse-Geisser |  | 174.486 |  | 117.12 |  | 1.490 |  |  |  |  |  |
| Task ✻ ProbeResponse ✻ Region |  | None |  | 6.796 |  | 2 |  | 3.398 |  | 1.336 |  | 0.270 |  |
|  |  | Greenhouse-Geisser |  | 6.796 |  | 1.40 |  | 4.850 |  | 1.336 |  | 0.265 |  |
| Task ✻ ProbeResponse ✻ Region ✻ FirstTask |  | None |  | 5.427 |  | 2 |  | 2.714 |  | 1.067 |  | 0.350 |  |
|  |  | Greenhouse-Geisser |  | 5.427 |  | 1.40 |  | 3.873 |  | 1.067 |  | 0.331 |  |
| Residual |  | None |  | 178.059 |  | 70 |  | 2.544 |  |  |  |  |  |
|  |  | Greenhouse-Geisser |  | 178.059 |  | 49.04 |  | 3.631 |  |  |  |  |  |
| Task ✻ ProbeResponse ✻ Laterality |  | None |  | 0.685 |  | 2 |  | 0.343 |  | 0.481 |  | 0.620 |  |
|  |  | Greenhouse-Geisser |  | 0.685 |  | 1.51 |  | 0.455 |  | 0.481 |  | 0.568 |  |
| Task ✻ ProbeResponse ✻ Laterality ✻ FirstTask |  | None |  | 3.229 |  | 2 |  | 1.614 |  | 2.264 |  | 0.111 |  |
|  |  | Greenhouse-Geisser |  | 3.229 |  | 1.51 |  | 2.142 |  | 2.264 |  | 0.126 |  |
| Residual |  | None |  | 49.913 |  | 70 |  | 0.713 |  |  |  |  |  |
|  |  | Greenhouse-Geisser |  | 49.913 |  | 52.76 |  | 0.946 |  |  |  |  |  |
| Task ✻ Region ✻ Laterality |  | None |  | 3.563 |  | 4 |  | 0.891 |  | 1.347 |  | 0.256 |  |
|  |  | Greenhouse-Geisser |  | 3.563 |  | 2.93 |  | 1.217 |  | 1.347 |  | 0.264 |  |
| Task ✻ Region ✻ Laterality ✻ FirstTask |  | None |  | 1.240 |  | 4 |  | 0.310 |  | 0.469 |  | 0.759 |  |
|  |  | Greenhouse-Geisser |  | 1.240 |  | 2.93 |  | 0.424 |  | 0.469 |  | 0.700 |  |
| Residual |  | None |  | 92.590 |  | 140 |  | 0.661 |  |  |  |  |  |
|  |  | Greenhouse-Geisser |  | 92.590 |  | 102.47 |  | 0.904 |  |  |  |  |  |
| ProbeResponse ✻ Region ✻ Laterality |  | None |  | 2.395 |  | 4 |  | 0.599 |  | 1.905 |  | 0.113 |  |
|  |  | Greenhouse-Geisser |  | 2.395 |  | 3.66 |  | 0.654 |  | 1.905 |  | 0.120 |  |
| ProbeResponse ✻ Region ✻ Laterality ✻ FirstTask |  | None |  | 2.217 |  | 4 |  | 0.554 |  | 1.763 |  | 0.140 |  |
|  |  | Greenhouse-Geisser |  | 2.217 |  | 3.66 |  | 0.606 |  | 1.763 |  | 0.146 |  |
| Residual |  | None |  | 44.010 |  | 140 |  | 0.314 |  |  |  |  |  |
|  |  | Greenhouse-Geisser |  | 44.010 |  | 128.15 |  | 0.343 |  |  |  |  |  |
| Task ✻ ProbeResponse ✻ Region ✻ Laterality |  | None |  | 3.080 |  | 4 |  | 0.770 |  | 1.459 |  | 0.218 |  |
|  |  | Greenhouse-Geisser |  | 3.080 |  | 3.35 |  | 0.919 |  | 1.459 |  | 0.226 |  |
| Task ✻ ProbeResponse ✻ Region ✻ Laterality ✻ FirstTask |  | None |  | 2.868 |  | 4 |  | 0.717 |  | 1.359 |  | 0.251 |  |
|  |  | Greenhouse-Geisser |  | 2.868 |  | 3.35 |  | 0.855 |  | 1.359 |  | 0.257 |  |
| Residual |  | None |  | 73.867 |  | 140 |  | 0.528 |  |  |  |  |  |
|  |  | Greenhouse-Geisser |  | 73.867 |  | 117.35 |  | 0.629 |  |  |  |  |  |
| Note. Type 3 Sums of Squares | | | | | | | | | | | | | |
|  | | | | | | | | | | | | | |
